# Survival After Combining Stereotactic Body Radiation Therapy and Tyrosine Kinase Inhibitors in Patients With Metastatic Renal Cell Carcinoma

**DOI:** 10.3389/fonc.2021.607595

**Published:** 2021-02-22

**Authors:** Yang Liu, Zhiling Zhang, Hui Han, Shengjie Guo, Zhuowei Liu, Mengzhong Liu, Fangjian Zhou, Pei Dong, Liru He

**Affiliations:** ^1^Department of Radiation Oncology, Sun Yat-sen University Cancer Center, State Key Laboratory of Oncology in South China, Collaborative Innovation Center for Cancer Medicine, Guangzhou, China; ^2^Department of Urology, Sun Yat-sen University Cancer Center, State Key Laboratory of Oncology in South China, Collaborative Innovation Center for Cancer Medicine, Guangzhou, China

**Keywords:** renal cell carcinoma, stereotactic body radiotherapy, tyrosine kinase inhibitors, survival, metastasis

## Abstract

**Background:**

Stereotactic body radiation therapy (SBRT) and tyrosine kinase inhibitors (TKIs) are effective treatments for metastatic renal cell carcinoma, but data on combining these two modalities are scarce. We aimed to investigate the survival outcomes of SBRT plus TKIs.

**Methods:**

Data of patients treated with TKIs from December 2007 to June 2019 were collected. Patients received SBRT plus TKIs (TKI + SBRT group) or TKIs alone (TKI alone group). Local control (LC), time to change of systemic therapy (TTS), and overall survival (OS) were assessed.

**Results:**

A total of 190 patients were included, and 85 patients received TKI + SBRT. The 2-year LC rate was 92.8%. The median OS in the TKI + SBRT group was significantly longer than that of the TKI alone group (63.2 vs 29.8 months; P < 0.001). In multivariate analysis, IMDC intermediate (HR 1.96; 95% CI 1.10–3.48; P = 0.022) and poor risk (HR 2.43; 95% CI 1.25–4.75; P = 0.009), oligometastasis (HR 0.41; 95% CI 0.26–0.65; P < 0.001), and the addition of SBRT (HR 0.48; 95% CI 0.31–0.75; P = 0.001) were prognostic factors for OS. Patients with oligometastasis (P = 0.009) and those with IMDC favorable (P = 0.044) or intermediate (P = 0.002) risk had significantly longer OS with TKI + SBRT. The median TTS were 21.5, 6.4, and 9.0 months in patients receiving SBRT before first-line TKI failure, SBRT after first-line TKI failure, and first-line TKI alone (P < 0.001). Five patients (5.9%) experienced SBRT-related grade 3 toxicities.

**Conclusions:**

Combining SBRT with TKIs is tolerable and associated with longer OS in selected patients, such as those with oligometastasis and favorable or intermediate risk.

## Introduction

Renal cell carcinoma accounted for 403,262 new cases worldwide in 2018 (1). Approximately 30%–40% of patients present with metastatic renal cell carcinoma (mRCC) (2). Targeted therapy has prolonged the survival of mRCC patients, yet the objective response rate (ORR) is low, ranging from 10%–30% (3, 4). Although the combination of immune checkpoint inhibitors and targeted therapy has substantially raised the ORR to about 40%–60% and prolonged the progression-free survival (PFS) to 12–15 months, complete response remains low, at less than 10% (5–7). In most cases, resistance to systemic agents is inevitable, and the depletion of effective systemic agents is merely a matter of time. Thus, systemic therapy requires other complementary treatment modalities to make additional gains in survival.

Metastasis-directed local therapy represents an indispensable component of mRCC treatment. Evidence on metastasis-directed surgery has demonstrated that the overall survival (OS) after complete metastasectomy is about 40.8 months, compared with 14.8 months after incomplete or no metastasectomy (8). In the era of targeted therapy, the importance of metastasectomy has somewhat decreased (9). On the one hand, perioperative targeted therapy application is associated with an increase in surgical complications (10). On the other hand, perioperative interruption of targeted therapy can result in rapid angiogenesis, which stimulates tumor growth (11).

Stereotactic body radiation therapy (SBRT) enables the delivery of intensified radiation doses in a highly conformal way, which could overcome the inherent radioresistance of renal cell carcinoma. The local control (LC) rate is around 90% after SBRT in mRCC (12), and deferred use or even permanent discontinuation of systemic therapy has been observed in oligometastasis patients receiving SBRT to all metastases (13, 14). Given the favorable therapeutic ratio of SBRT in mRCC, the current National Comprehensive Cancer Network guidelines have recommended it as an effective treatment option for oligometastases.

Current studies on patients with mRCC have predominantly focused on the role of SBRT alone in oligometastatic or oligoprogressive settings (13, 15). A few studies investigating the combined use of tyrosine kinase inhibitors (TKIs) and SBRT have only reported the results of response rates and local control (16, 17). Considering the lack of evidence regarding the survival gains obtained by adding SBRT to TKI treatment in patients with mRCC, our study aimed to compare the survival outcomes of patients receiving SBRT plus TKIs versus TKIs alone.

## Materials and Methods

### Patients

This study was approved by our institutional review board (ID: B2020-057-01), and informed consent was waived. We retrospectively reviewed the medical records of patients with mRCC treated in our center between December 2007 and June 2019. Eligible patients were aged ≥ 18 years who received TKI treatment for mRCC. Those who were followed up for less than three months, were treated with conventionally fractionated radiotherapy, or received immunotherapy as first-line treatment were excluded.

### Treatment

Usually, patients were recommended to initiate TKI treatment shortly after the diagnosis of mRCC. The TKIs were administered at their usual dosage regimens in accordance with current guidelines. No interruption or dose reduction of TKI was required during SBRT, except for serious treatment-related toxicities.

SBRT was delivered to all lesions in oligometastasis, to the major tumor burden or oligoprogressive lesions as cytoreductive therapy, and to the symptomatic lesions with palliative intent. Oligometastasis and oligoprogression were defined as the presence of no more than five metastatic and progressive sites, respectively, without brain or liver involvement. Major tumor burden was defined as the largest lesion accounting for at least 50% of the tumor burden, which was calculated as the sum of the longest unidimensional diameter of the target lesions as per Response Evaluation and Criteria in Solid Tumours (RECIST) version 1.1.

All patients underwent computed tomography (CT) with or without magnetic resonance imaging simulation scanning with site-specific immobilization as previously described. Four-dimensional CT was applied to the lungs and used for some upper abdominal lesions. In all patients, SBRT was implemented with volumetric intensity modulated arc therapy planning. SBRT was predominantly delivered in five fractions, either once daily or every other day. The biologically effective dose (BED) was calculated using the linear-quadratic model, with (18). Cone beam CT was performed before every treatment.

### Outcomes

Clinical examination and follow-up scans were recommended every three months for the first two years. The response of bone metastases to SBRT was evaluated according to the University of Texas MD Anderson Cancer Center criteria (19). Otherwise, response was evaluated according to RECIST version 1.1. OS was defined from the time of metastasis detection to the last follow-up or death. Time to change of systemic therapy (TTS) was calculated from the start of first-line TKIs to the initiation of second-line therapy. PFS after SBRT was calculated from the start of SBRT to disease progression or death. LC was defined as freedom from progression at the treated sites after SBRT. Toxicities were graded according to the Common Terminology Criteria for Adverse Events 4.0 rating scale.

### Statistical Analysis

Categorical data were compared using the chi-squared test, and continuous variables were compared by Mann-Whitney tests. The Kaplan–Meier method and log-rank test were used to estimate and compare survival among the groups, respectively. The Cox regression method was used to analyze the hazard ratios (HRs) and associated 95% confidence intervals (CIs) for OS. Univariate and multivariate analyses were performed, and only the factors evaluated as significant in the univariate analyses were included in the multivariate model. A two-sided P-value of < 0.05 was considered statistically significant. SPSS version 23 (IBM Corp., Armonk, NY, USA) was used for statistical analyses.

## Results

### Patient and Treatment Characteristics

In total, 190 mRCC patients treated with TKIs were identified. Eighty-five patients (44.7%) received SBRT in addition to TKIs (TKI + SBRT group), while 105 patients (55.3%) were treated with TKIs alone (TKI alone group). At the time of metastasis detection, 82 patients (43.2%) had oligometastasis. One-hundred and forty-nine patients (78.4%) had intermediate or poor risk, according to the International Metastatic Renal Cell Carcinoma Database Consortium (IMDC) criteria. Baseline characteristics were similar between the TKI + SBRT and TKI alone groups, except that patients in the TKI + SBRT group were older and were more likely to have bone metastases (**Table 1**).

**Table 1:** Baseline characteristics (N=190).

Characteristics, N (%)	Total (N=190)	TKI Alone (N=105)	TKI + SBRT (N=85)	*P*
Age, median (range), years	54 (18–86)	54 (18–83)	55 (21–86)	0.049
Gender				0.666
Male	147 (77.4)	80 (76.2)	67 (78.8)	
Female	43 (22.6)	25 (23.8)	18 (21.2)	
Pathology				0.125
Clear cell	140 (73.7)	82 (78.1)	58 (68.2)	
Non-clear cell	50 (26.3)	23 (21.9)	27 (31.8)	
IMDC risk group				0.412
Favorable	41 (21.6)	23 (21.9)	18 (21.2)	
Intermediate	110 (57.9)	57 (54.3)	53 (62.3)	
Poor	39 (20.5)	25 (24.8)	14 (16.5)	
Metastatic sites				
Lung	90 (47.4)	53 (50.5)	37 (43.5)	0.340
Bone	66 (34.7)	20 (19.0)	46 (54.1)	<0.001
Liver	18 (9.5)	13 (12.3)	5 (5.9)	0.128
Brain	8 (4.2)	6 (5.7)	2 (2.4)	0.433
Synchronous metastasis				0.485
Yes	97 (51.1)	56 (53.3)	41 (48.2)	
No	93 (48.9)	49 (46.7)	44 (51.8)	
Oligometastasis				0.204
Yes	82 (43.2)	41 (39.0)	41 (48.2)	
No	108 (56.8)	64 (61.0)	44 (51.8)	
Nephrectomy				0.103
Yes	159 (83.7)	92 (87.6)	67 (78.8)	
No	31 (16.3)	13 (12.4)	18 (21.2)	

Sunitinib was the most common first-line systemic therapy, accounting for 57.9% of the cases. Fifteen patients (7.9%) discontinued first-line TKI because of intolerable toxicities despite dose-schedule adjustments, leaving 175 patients treated with first-line TKI regularly. A total of 144 lesions were treated with SBRT. SBRT was indicated for oligometastasis in 28 patients (32.9%), oligoprogression in eight patients (9.4%), major tumor burden in 16 patients (18.8%), and palliation in 33 patients (38.8%). Nearly 70% of the irradiated sites were located in the bones. One-hundred and eighteen lesions (81.9%) received 35–45 Gy in five fractions, and the median BED_3_ of all irradiated sites was 146.7 Gy (65.6–237.5 Gy) (**Supplementary Table 1**).

### Response to SBRT

Complete response, partial response, stable disease, and progressive disease (PD) were recorded in 30 (20.8%), 89 (61.8%), 22 (15.2%), and 3 (2.1%) sites after SBRT, resulting in an ORR of 82.6%. With a median follow-up of 13.6 months after SBRT, PD was observed in three lesions after SBRT. The 1-year and 2-year LC rates were 99.2% and 92.8%, respectively.

### Survival and Prognostic Factors

**Figure 1:**
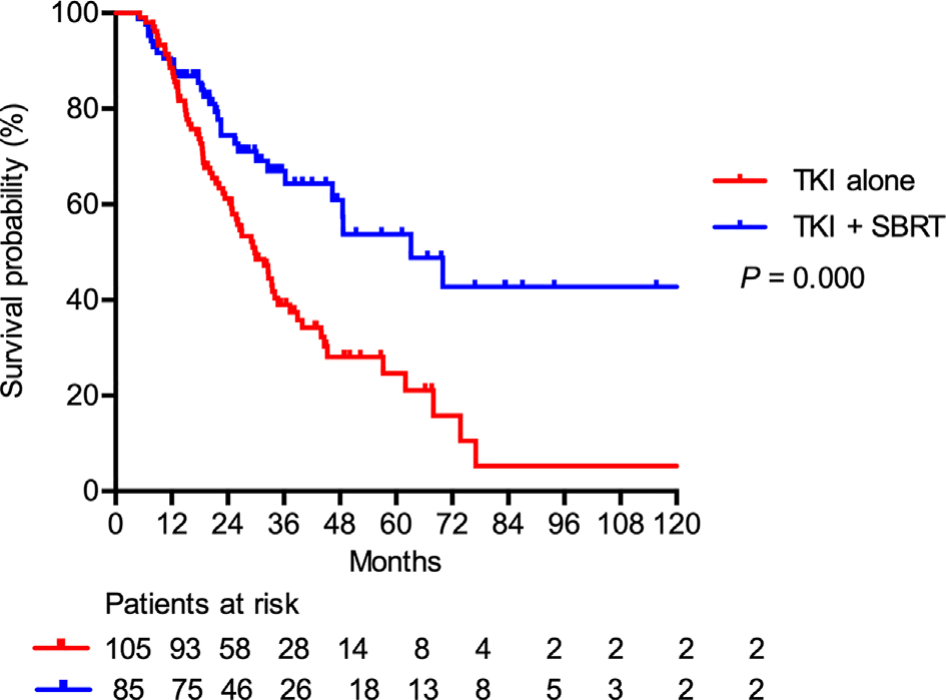
Overall survival in patients treated with stereotactic body radiation therapy (SBRT) in addition to tyrosine kinase inhibitors (TKI) (TKI + SBRT group) and TKI alone.

At a median follow-up of 25.8 months (range, 4.8–122.7 months), nine patients (4.7%) were lost to follow-up and 86 patients (45.3%) were still alive. The median PFS after SBRT was 9.0 months. For the entire cohort, the median OS was 36.3 months. The median OS was significantly longer in the TKI + SBRT group than in the TKI alone group (63.2 vs 29.8 months; P < 0.001). The OS rates at 2 and 5 years were 74.4% and 53.8% in the TKI + SBRT group and 61.2% and 24.6% in the TKI alone group, respectively (**Figure 1**).

**Table 2** summarizes the results of univariate and multivariate analyses. In the multivariate analysis, intermediate (HR 1.96; 95% CI 1.10–3.48; P = 0.022) and poor IMDC risk groups (HR 2.43; 95% CI 1.25–4.75; P = 0.009) were associated with inferior OS, whereas oligometastasis (HR 0.41; 95% CI 0.26–0.65; P < 0.001) was correlated with good prognosis. The addition of SBRT was associated with a 52% decreased hazard of death (HR 0.48; 95% CI 0.31–0.75; P = 0.001).

**Table 2:** Prognostic factors of overall survival (N=190).

Variables	Univariate analysis		Multivariate analysis	
	HR (95% CI)	*P*	HR (95% CI)	*P*
Oligometastasis				
No	Reference		Reference	
<Yes	0.36 (0.23, 0.57)	<0.001	0.41 (0.26, 0.65)	<0.001
Nephrectomy				
No	Reference		Reference	
Yes	0.49 (0.29, 0.84)	0.009	0.65 (0.37, 1.12)	0.120
Treatment				
TKI alone	Reference		Reference	
TKI + SBRT	0.46 (0.30, 0.72)	0.001	0.48 (0.31, 0.75)	0.001
IMDC score				
Favorable	Reference		Reference	
Intermediate	2.08 (1.17, 3.69)	0.012	1.96 (1.10, 3.48)	0.022
Poor	3.59 (1.87, 6.89)	<0.001	2.43 (1.25, 4.75)	0.009

**Figure 2:**
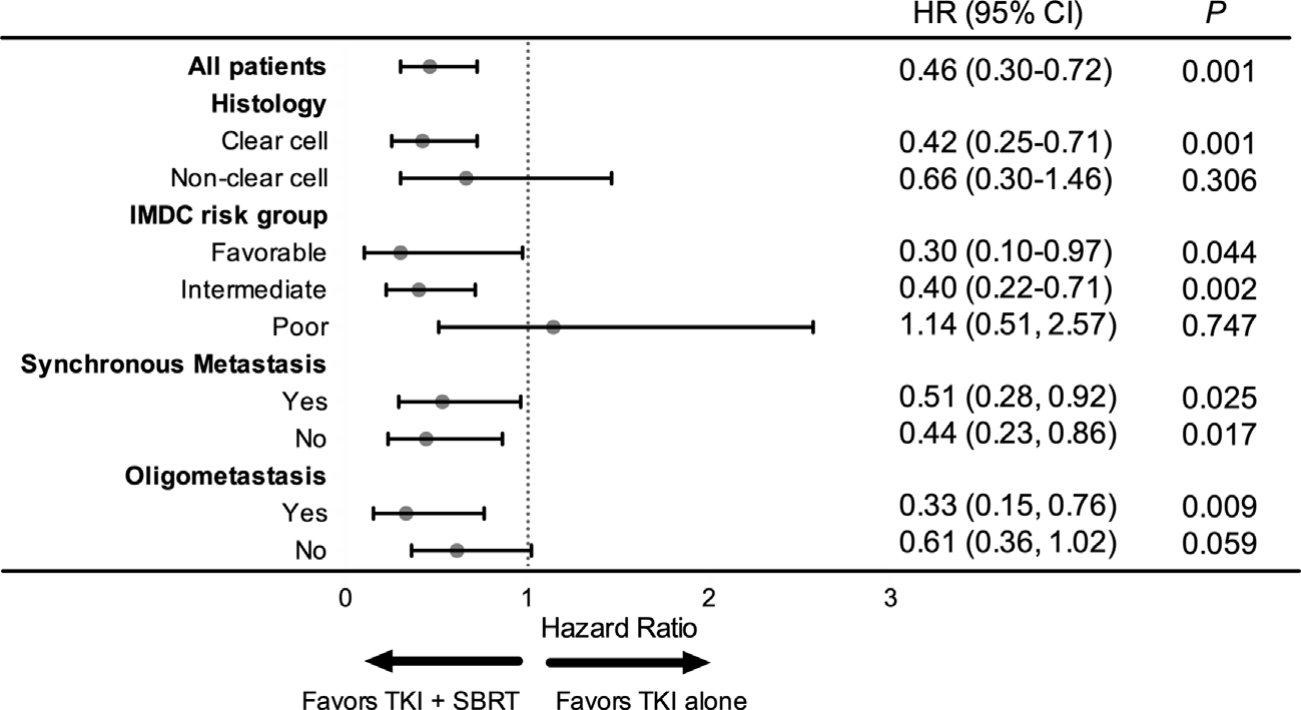
Forest plot of the association between tyrosine kinase inhibitors (TKI) + stereotactic body radiation therapy (SBRT) and overall survival by subgroup. HR, hazard ratio; CI, confidential interval.

**Figure 3:**
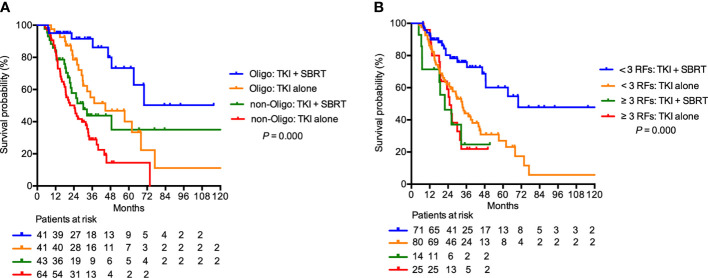
Overall survival in patients treated with stereotactic body radiation therapy (SBRT) in addition to tyrosine kinase inhibitors (TKI) (TKI + SBRT group) and TKI alone stratified by **(A)** oligometastasis and **(B)** International Metastatic Renal Cell Carcinoma Database Consortium (IMDC) risk factors. IMDC favorable or intermediate risk group were illustrated as < 3 RFs, and poor risk group was illustrated as ≥ 3 RFs. RFs, risk factors; Oligo, oligometastasis.

In the subgroup analysis, patients with clear cell histology (P = 0.001), IMDC favorable (P = 0.044) and IMDC intermediate risk group (P = 0.002), and oligometastasis (P = 0.009) had significant improvement in OS after adding SBRT (**Figure 2**). Patients with oligometastasis who received TKI + SBRT treatment have the most favorable outcome, with median OS not reached (P < 0.001; **Figure 3A**). As for subgroups stratified by the IMDC criteria, the median OS in the TKI + SBRT and TKI alone groups were 70.0 months and 33.3 months in the favorable or intermediate risk group and 21.9 months and 25.0 months in the poor risk group, respectively (P < 0.001; **Figure 3B**).

### SBRT Delivered With First-Line TKI

**Figure 4:**
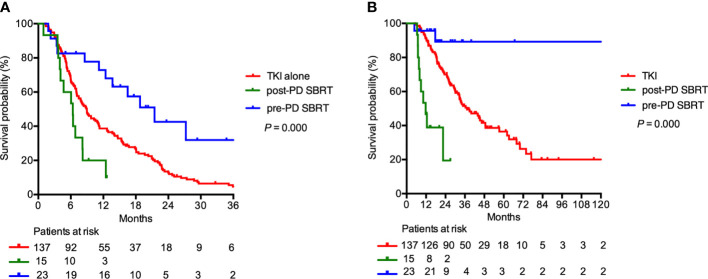
**(A)** Time to change of systemic therapy and **(B)** overall survival in patients receiving regular first-line TKI treatment (N=175). Patients may receive stereotactic body radiation therapy (SBRT) before first-line tyrosine kinase inhibitors (TKI) failure (pre-PD SBRT), SBRT after first-line TKI failure (post-PD SBRT), or first-line TKI alone.

In the 175 patients receiving regular first-line TKI treatment, SBRT was delivered concomitantly with first-line TKI treatment to 38 patients (21.7%). Among them, 23 patients (60.5%) underwent irradiation before first-line TKI failure (pre-PD SBRT group), and the remaining 15 patients (39.5%) received SBRT after first-line TKI failure (post-PD SBRT group). For the entire subgroup, the median TTS after first-line TKIs was 9.0 months. The median TTS after first-line TKIs was similar in patients treated with first-line TKI with or without SBRT (12.4 vs 9.0 months; P = 0.139). However, the median TTS were 21.5 months, 6.4 months, and 9.0 months in the pre-PD SBRT, post-PD SBRT, and first-line TKI alone groups (P < 0.001; **Figure 4A**). The OS was longer in the pre-PD SBRT group than in the post-PD SBRT or first-line TKI alone groups (median OS not reached vs 11.2 vs 39.3 months, 2-year OS 89.3% vs 19.4% vs 70.0%; P < 0.001; **Figure 4B**).

### Toxicities After SBRT

SBRT combined with TKI was generally well tolerated. No grade 4 or 5 toxicities occurred. Grade 3 and grade 2 toxicities were reported in five patients (5.9%) and 24 patients (28.2%), respectively. There were 10 events of grade 3 toxicities, eight (80.0%) of which were hematological toxicities that were later resolved (**Table 3**). The number of SBRT-related toxicities were similar between the pre-PD SBRT group and the post-PD SBRT group. The number of grade 1, 2, and 3 SBRT-related events were 8, 1, and 2 in the pre-PD SBRT group, and 3, 3, and 2 in the post-PD SBRT, respectively.

**Table 3:** Radiotherapy-related toxicity.

	Grade 1	Grade 2	Grade 3
Dermatitis radiation	4	2	1
Alopecia	1		
Skin induration	1		
Nausea/Vomiting	16	4	
Colonic hemorrhage		1	
Neuropathy	3	3	1
Pneumonitis	6		
Bronchopleural fistula		2	
Neutropenia	13	9	2
Anemia	9	2	6
Thrombocytopenia	3	1	
Fracture	7	2	

## Discussion

Although a couple of studies have validated the safety of combining SBRT with TKIs (16, 17, 20), the impact of SBRT on survival remains unknown. Our study demonstrated that the integration of SBRT and TKIs was associated with improved survival compared with that with TKIs alone in patients with mRCC. To our knowledge, this study represents the largest report on patients’ survival after SBRT plus TKIs in the general mRCC patient population.

In our study, the median OS of patients in the TKI alone group was similar to that reported in the studies of sequential targeted therapies (median OS, 18–30 months) (21). The addition of SBRT to TKI was associated with significant reduction in the hazard of death. Preclinical studies suggest that combining SBRT and TKIs might yield superior anti-tumor activity. TKIs may enhance the tumor response to SBRT through several mechanisms, including the reversal of hypoxia in the tumor microenvironment, the facilitation of apoptosis, and the prevention of SBRT-induced re-vascularisation (22, 23). SBRT could potentiate the effect of TKIs by eradicating resistant clones, destroying the tumor microvasculature, inhibiting growth factors and inducing an anti-tumor immune response (12, 24). As observed in some clinical studies, concurrent TKI treatment and SBRT is safe might achieve a superior tumor response (16, 17, 20). The results of our study imply that beyond tumor response improvement, SBRT may be associated with improved survival in some patients. However, whether the survival benefit is truly realized by the addition of SBRT needs to be verified in prospective trials.

In addition to the reports of survival, our study provided potential insights into patient selection for combined modality therapy. Our cohort observed that the addition of SBRT was associated with better survival among patients with oligometastasis or those with favorable or intermediate risk. Oligometastasis has been generally accepted as an indicator for local therapy. In studies that included oligometastatic mRCC patients, the median OS of patients treated with SBRT was remarkable (median OS, 34–51 months) (14, 25, 26), with some not even reaching the median OS (13, 27). The patient’s IMDC risk group may also be an indicator for treatment selection. In the update on the CARMENA trial, patients with more than one risk factor according to the IMDC criteria did not benefit from cytoreductive nephrectomy (28), which was similar to our findings. In the favorable or intermediate risk groups, however, we observed a significantly longer OS in patients in the TKI + SBRT group than in the TKI alone group. These results suggest that the addition of local therapy may be beneficial for the subgroups of patients with favorable prognosis, such as those with oligometastasis, and IMDC favorable or intermediate risk.

Finally, our study may provide some clues as to the sequence in which the systemic and local therapies should be administered. Our results showed that mRCC patients treated with SBRT before first-line TKI failure had better survival than those who received SBRT after first-line TKI failure. The traditional concept of upfront cytoreductive nephrectomy has been reshaped in the era of targeted therapy. In the CARMENA trial and the SURTIME trial, upfront cytoreductive nephrectomy failed to demonstrate survival gains over sunitinib alone, but survival benefit was observed in the deferred nephrectomy arm (28, 29). Besides, patients with early disease progression during first-line sunitinib had a similarly poor prognosis, regardless of when nephrectomy was implemented (30). These results imply that local therapy may still have a role in mRCC management, and attention should be paid to the sequence of different therapies (9). Targeted therapy followed by local therapy may be a more effective strategy, as initial targeted therapy may be able to screen out patient tumors with aggressive biological behavior that demand intensification of systemic therapy instead of local therapy.

Our study has several limitations. Firstly, it is retrospective. Patients included in the SBRT group may represent a selected cohort with indolent disease that could not be fully elucidated by current clinical parameters. Secondly, SBRT was delivered at various timepoints for different purposes. Thirdly, we cannot control for the type and sequence of targeted regimens. Finally, our study was conducted in a high-volume cancer center, and these results might be difficult to replicate in smaller centers. Future studies with multiple centers involved could reduce this selection bias, especially when standardized data collection and retrieval project has been designed (31).

## Conclusions

Our study suggests that the use of SBRT on top of current TKI treatment is tolerable and may be associated with survival improvement. Patients with oligometastasis and with favorable or intermediate risk as per the IMDC criteria may be potential candidates for this combined modality treatment. The value of local therapy may be diminished in patients who progress during first-line systemic therapy. Prospective studies are needed to confirm our findings and determine the candidates, the timing of implementation, and the optimal combining strategy of the two treatments.

## Data Availability Statement

The raw data supporting the conclusions of this article will be made available by the authors, without undue reservation.

## Ethics Statement

The studies involving human participants were reviewed and approved by the Ethical Committee of Sun Yat-Sen University Cancer Center. Written informed consent for participation was not required for this study in accordance with the national legislation and the institutional requirements.

## Author Contributions

YL, PD, and LH contributed to the conception and design. ZZ, HH, SG, ZL, ML, FZ, and PD contributed to data collection. YL performed data analysis and interpretation. YL and ZZ contributed to manuscript preparation. LH and PD revised the manuscript. All authors contributed to the article and approved the submitted version.

## Conflict of Interest

The authors declare that the research was conducted in the absence of any commercial or financial relationships that could be construed as a potential conflict of interest.
