# Supplementary material for: Survival After Combining Stereotactic Body Radiation Therapy and Tyrosine Kinase Inhibitors in Patients With Metastatic Renal Cell Carcinoma
Source: Front Oncol. 2021 Feb 22;11:607595. doi: 10.3389/fonc.2021.607595 (PMC7937906; doi:10.3389/fonc.2021.607595)
Supplement: Supplementary file 1 [file Table_1.docx]

Supplementary Material

Supplementary Table 1. Characteristics of irradiated sites (N=144)

| Irradiated sites | N (%) |
| --- | --- |
| Lung | 8 (5.6) |
| Bone | 98 (68.1) |
| Lymph node | 10 (6.9) |
| Soft tissue | 5 (3.5) |
| Brain | 3 (2.1) |
| Adrenal gland | 5 (3.5) |
| Kidney | 9 (6.3) |
| Others | 6 (4.2) |
| Prescription dose |  |
| 16 Gy /1 fx | 1 (0.7) |
| 20 Gy/1 fx | 1 (0.7) |
| 24 Gy/3 fx | 1 (0.7) |
| 27 Gy/3 fx | 1 (0.7) |
| 30 Gy/5 fx | 5 (3.5) |
| 35 Gy/5 fx | 23 (16.0) |
| 36 Gy/ 5 fx | 4 (2.8) |
| 38 Gy/ 5 fx | 5 (3.5) |
| 40 Gy/5 fx | 61 (42.4) |
| 42 Gy/ 5 fx | 3 (2.1) |
| 43 Gy/ 5 fx | 5 (3.5) |
| 45 Gy/5 fx | 17 (11.8) |
| Others | 17 (11.8) |
